# Humane stunning or stun/killing in the slaughter of wild-caught finfish: The scientific evidence base

**DOI:** 10.1017/awf.2023.30

**Published:** 2025-05-29

**Authors:** Katy L James, Salvador Prats Aparicio, Nilantha S Jayasuriya, Tharangani K Herath, Jeff Lines, Lynne U Sneddon, Upali S Amarasinghe, Nicola P Randall

**Affiliations:** 1 Harper Adams University, Newport, Shropshire TF10 8NB, UK; 2 Silsoe Livestock Systems Ltd, Wrest Park, Silsoe, Bedfordshire, MK45 4HP, UK; 3 University of Gothenburg, Department of Biological & Environmental Sciences, 40530 Göteborg, Sweden; 4 Department of Zoology and Environmental Management, University of Kelaniya, Kelaniya, 11600, Sri Lanka

**Keywords:** animal welfare, electrical stunning, fish welfare, flesh quality, percussive stunning, wild-caught fish slaughter

## Abstract

An estimated 0.9 to 2.5 trillion individual wild finfish, destined for human or animal consumption, are captured globally each year by commercial fisheries. The majority of wild-caught fish die either from asphyxiation or evisceration; slaughter methods considered inhumane. Humane stunning or stun/killing may improve the welfare of wild-caught fish at the time of slaughter by rendering fish immediately insensible to pain, stress and suffering. Reducing fish stress during slaughter may also improve product quality. This study systematically mapped and narratively evaluated scientific research regarding humane stunning or stun/killing of wild finfish caught for food and feed on a commercial scale. Multiple knowledge gaps were identified: Key humane stunning parameters have only been scientifically tested, in terms of fish welfare and/or flesh quality, for a minority of wild-caught fish species and stunning methods. More species-specific protocols and use of brain activity measurements to accurately assess state of consciousness on application of the stunning method are required. More scientific testing of stunning methods in commercial wild-capture settings is needed to validate findings of controlled laboratory studies and ensure the methods are practical in fisheries. Research is needed to better understand the potential economic benefits of humane stunning for fishermen. From a donor perspective this review highlights the need to support more primary research into the feasibility and implementation of humane stunning or stun/killing in wild-capture fisheries.

## Introduction

An estimated 0.9 to 2.5 trillion individual wild finfish are captured globally each year by commercial fisheries and destined for human or animal consumption. This estimate does not include discards or fish caught illegally, and so the numbers are likely to be higher.

Scientific research supports the consensus that fish are sentient beings (European Food Safety Authority [EFSA] 2009a; Braithwaite 2010; Broom 2016; Sneddon & Brown 2020). The majority of wild-caught fish that are alive when landed die either from asphyxiation (in air or ice) or evisceration (Robb & Kestin 2002; Anders *et al.* 2019). These slaughter methods result in poor fish welfare because they do not cause immediate insensibility to pain, stress and suffering at the time of killing (EFSA 2004).

Based on scientific research, both the EFSA and the World Organisation for Animal Health (OIE) recommend that farmed fish are stunned prior to slaughter using a method that induces immediate unconsciousness or instantaneous death (stun/killing), and if unconsciousness is reversible fish should be killed before consciousness is recovered (EFSA 2004; OIE 2019). Unconsciousness is defined by EFSA as “a state of unawareness [loss of consciousness] in which there is temporary or permanent damage to brain function and the individual is unable to respond to normal stimuli, including pain” (EFSA 2004). Stunning methods that cause instantaneous death or that induce immediate unconsciousness aim to minimise poor fish welfare at time of slaughter by ensuring that the animal is insensible to fear, anxiety, pain, suffering and distress (EFSA 2004). This is often referred to as humane stunning or stun/killing (e.g. EFSA 2004). Humane stunning or stun/killing methods recommended for farmed fish fall into two main categories, mechanical, including percussive stunning, spiking or coring and free bullet, and electrical (in-water, semi-dry and dry) (EFSA 2009[b]-[h]; OIE 2019). In general, mechanical stunning, if applied correctly, is irreversible, whereas consciousness may be regained following electrical stunning (OIE 2019).

Humane stunning is now routinely used in some aquaculture industries, notably rainbow trout (*Oncorhynchus mykiss*) and Atlantic salmon (*Salmo salar*), albeit in a small number of, mostly European, countries (Humane Slaughter Association [HSA] 2018). Moreover, in some countries, it is now a legal requirement to humanely stun farmed fish, for example Norway (Decree No 1,250 2006). To date, however, guidance and legislation for humane stunning or stun/killing of wild-caught fish is limited, with the exception of Swiss law, which is heavily caveated and in practice only for fishing from lakes (Animal Protection Ordinance 455.1 2008), and New Zealand law that only applies to wild finfish that are caught and held for killing at a later time (New Zealand’s Commercial Slaughter Code of Welfare 2018).

Pre-slaughter stress initiates behavioural and physiological responses in fish that negatively affect flesh quality (Robb & Kestin 2002). The potential synergy between improved welfare resulting from humane stunning and quality of product, is recognised in the aquaculture industry (Humane Slaughter Association 2018). Furthermore, automated stunning technology has in some cases led to labour cost savings on fish farms (van de Vis *et al*. 2017). The development of humane stunning technologies for aquaculture continues to be an active area of research (Papaharisis *et al.* 2019). Knowledge and technology from this sector are likely to be highly relevant to the development and promotion of humane stunning in wild-capture fisheries.

The aim of this review was to systematically catalogue and synthesise scientific research regarding humane stunning or stun/killing of finfish wild-caught for food and feed on a commercial scale. The review considered implications for fish welfare and flesh quality, as well as any economic, environmental, ethical and social considerations, including practicality. The work formed part of a project titled ‘Systematic review and feasibility study – stunning or killing of wild-caught fish in commercial fisheries’ funded by the Humane Slaughter Association. The scope of the review was set by the funder and aimed to provide a better understanding of the evidence base as well as identify knowledge gaps.

## Materials and methods

### A priori *protocol*

The methodology for collating, screening and cataloguing published and grey scientific research literature followed globally recognised guidelines for systematic mapping; a type of evidence synthesis particularly valuable for addressing broad topics that include multiple interventions, populations or outcomes (James *et al.* 2016; Collaboration for Environmental Evidence 2018). The methodology was detailed in an *a priori* protocol (James *et al.* 2020) that conformed to reporting standards for evidence syntheses in environmental research (ROSES) for systematic map protocols (Haddaway *et al.* 2017). A request for public comment on a draft version of the protocol was made between 26th August and 9th October 2020, and edits made in response to comments received. This review followed the methodology outlined in the protocol but included literature in Spanish as well as English. In addition to the systematic mapping process, a narrative evaluation of the included studies was included. A brief overview of the methodology is provided below.

### Searches for evidence

A comprehensive search of seven academic bibliographic databases and platforms (including the first 500 hits from Google Scholar after sorting for relevance) and 20 websites of key organisations, was carried out to capture published and unpublished grey literature. Relevant literature supplied by stakeholders was also included. The database/platforms searched were: Scopus; Food Science Source; CAB abstracts; Web of Science Core Collections; Electronic Theses Online Service (EThOS); Digital Access to Research Theses (DART-Europe E thesis). Specialist websites searched were: 1. Fish Count; 2. Centre for Environment Fisheries and Aquaculture Science 3. Defra online databases; 4. Food and Agriculture Organisation; 5. Universities Federation for Animal Welfare; 6. The Foundation for Industrial and Technical Research (SINTEF) Fisheries and Aquaculture; 7. World Wildlife Fund; 8. International Marine Ingredient Association; 9. Sea Fish; 10. WorldFish; 11. Marine Stewardship Council; 12. Compassion in World Farming; 13. European Food Safety Authority; 14. Wageningen University; 15. Norwegian Institute of Food, Fisheries and Aquaculture Research (Nofima); 16. Ace Aquactec Ltd; 17. Fair Fish International - FishEthobase; 18. Humane Slaughter Association; 19. European Commission; 20. Royal Society for the Protection of Animals.

A search string to capture literature from bibliographic databases was applied in both English and Spanish: (*Fish*) AND (stun* OR slaughter* OR welfare OR electronarcosis OR euthan* OR “electric shock”) NOT (stunt* OR pig* OR swine OR pork OR cattle OR cow* OR beef OR chicken* OR poultry OR turkey* OR lamb* OR sheep OR calf OR calves OR bull* OR jellyfish* OR crab* OR trematode*).

Where the use of Boolean search strings was not allowed, keywords were used to search sites. Searches of organisational websites were conducted in the English language only. Literature was captured between 20th October and 3rd November 2020 and a re-run of the searches was carried out between 10th and 15th February 2022 to update results. Detail of all searches conducted are provided in Appendix 1, worksheets 1–3.

### Article screening

Literature captured was imported into systematic review software (EPPI-Reviewer), and duplicate articles removed. Search results were screened for relevance against inclusion criteria (Table 1) in a two-stage process: (1) title and abstract (screened concurrently for efficiency); (2) full text. The reviewers had not authored any articles that were considered for inclusion. Every attempt was made to retrieve full texts, and any articles that could not be located or accessed were recorded (Appendix 1, worksheet 4). The number of articles included at each stage was recorded, as were reasons for exclusion of articles at full text (Appendix 1, worksheet 5).

**Table 1.** Eligibility criteria for study inclusion in the review

Eligibility criteria	Description
Populations	Wild finfish, caught on any commercial scale, from inland and marine waters, and intended for consumption by humans and/or animals. Recreational and subsistence fishing, were excluded. Any species or group of mixed species were considered. Studies from fish farms and laboratory-based studies on farmed fish deemed relevant to wild-caught fish (i.e. the species is caught on a commercial scale in the wild) were included
Stunning method	OIE (2019) recommended stunning or stun/kill methods for farmed fish for human consumption, which EFSA (2004, 2009[b]-[h]) regard as humane if applied correctly. These include, either alone or in combination: percussive stunning (mechanical or manual), spiking or coring, free bullet, and electrical stunning (dry, semi-dry, wet). Studies solely investigating killing methods shown to result in poor fish welfare (EFSA 2004, 2009[b]-[h]): chilling with ice in holding water, carbon dioxide (CO_2_) in holding water; chilling with ice and CO_2_ in holding water; salt or ammonia baths; asphyxiation by removal from water; exsanguination without stunning, were excluded. Studies that compared OIE (2019) recommended stunning or stun/kill methods and killing methods shown to result in poor fish welfare (EFSA 2004, 2009[b]-[h]) were included. Novel or modified stunning methods that are potentially humane but not yet recommended by the OIE (2019) were categorised separately. Studies that compared a recommended humane method with a potentially humane method, were included and meta-data extracted
Eligible comparators	No stunning; alternative stunning methods/devices including methods that the OIE (2019) consider to be inhumane; no comparator
Eligible outcomes	Outcomes were captured iteratively from the literature but included: the impact of the intervention on fish welfare; post-stunning flesh quality and any practical, economic, social, socio-economic, ethical or environmental implications resulting from stunning
Eligible data type	Quantitative and qualitative research, including both primary empirical research and secondary research (reviews catalogued in a separate database)
Eligible study design	Any primary empirical or secondary research. Meta-data was extracted from primary empirical research studies only. Reviews were categorised separately and screened for relevant studies to ensure that no primary empirical studies had been missed in the searches. Commentaries were excluded
Geographical limitations	None
Date restrictions	None
Language restrictions	English and Spanish language only

Prior to commencing screening at both stages, a random sub-set of 10% of articles were screened by the two reviewers and the level of agreement calculated using Cohen’s Kappa analysis (Landis & Koch 1977). The resulting Kappa statistic indicated substantial agreement (0.69) at title and abstract screening and near perfect agreement at full text level (0.84). All disagreements were discussed, and a consensus agreed upon.

### Database of scientific research

Metadata were coded for each primary empirical study from articles included at full text. The key variables were: (1) bibliographic information; (2) study characteristics; (3) study design; (4) population; (5) stunning/slaughter process; (6) parameters recorded in the experiment; and (7) outcomes reported. The full data coding strategy is provided in the protocol. Multiple studies reported within one article were considered separately. A ‘study’ was defined as an experiment: (1) with clearly different objectives to any other in the article; (2) where one species of fish was studied independently from any other in the article; (3) conducted in a different country to any other in the article. Meta-data extraction and coding were performed by two reviewers following consistency checking on an initial coding of subset of between 10% full texts, discussing all disagreements.

Reviews, screened for relevant primary research, and studies about potentially humane methods that are not yet recognised by the OIE (2019) were catalogued in separate databases and no meta-data were coded.

Primary research studies catalogued in the database were used to describe the volume, trends and characteristics of the evidence base and to identify where knowledge gaps lay. Study results were also narratively synthesised to better understand implications for: fish welfare; flesh quality and practical implementation of humane stunning or stun/killing in commercial practice.

## Results and Discussion

### Literature search and screening

**Figure 1.**
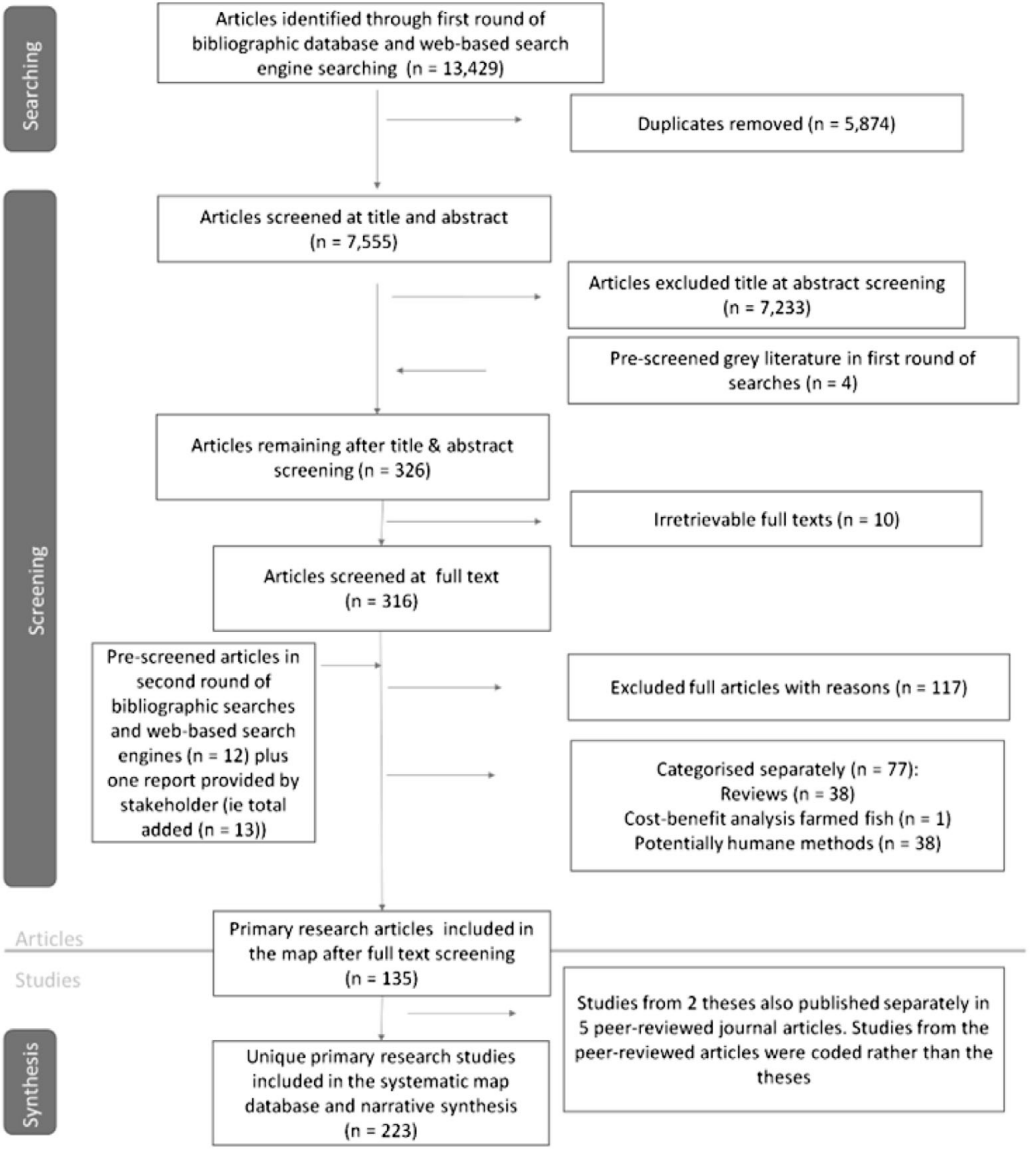
Flow diagram of literature included and excluded at each stage of the review process.

A total of 135 primary research articles were included in the review (Appendix 2, worksheet 1). Figure 1 shows a flow diagram summarising the number of articles included at each stage of the process. Two of the 135 articles were theses (Grimsbø 2016; Rucinque 2016) with relevant studies also published in peer-reviewed journals. To prevent ‘double coding’, studies from the peer-reviewed articles were coded rather than those from the theses. Therefore, the primary research database contained a total of 223 unique studies from 133 articles (Appendix 2, worksheet 2).

Seventy-seven articles were categorised in separate databases: 38 reviews (Appendix 2, worksheet 3); one report about cost benefit of stunning methods in aquaculture (Appendix 2, worksheet 3); 38 articles about novel stunning methods that are potentially humane but not yet recognised by the OIE (2019) (Appendix 2, worksheet 4).

### Publication year and type

**Figure 2.**
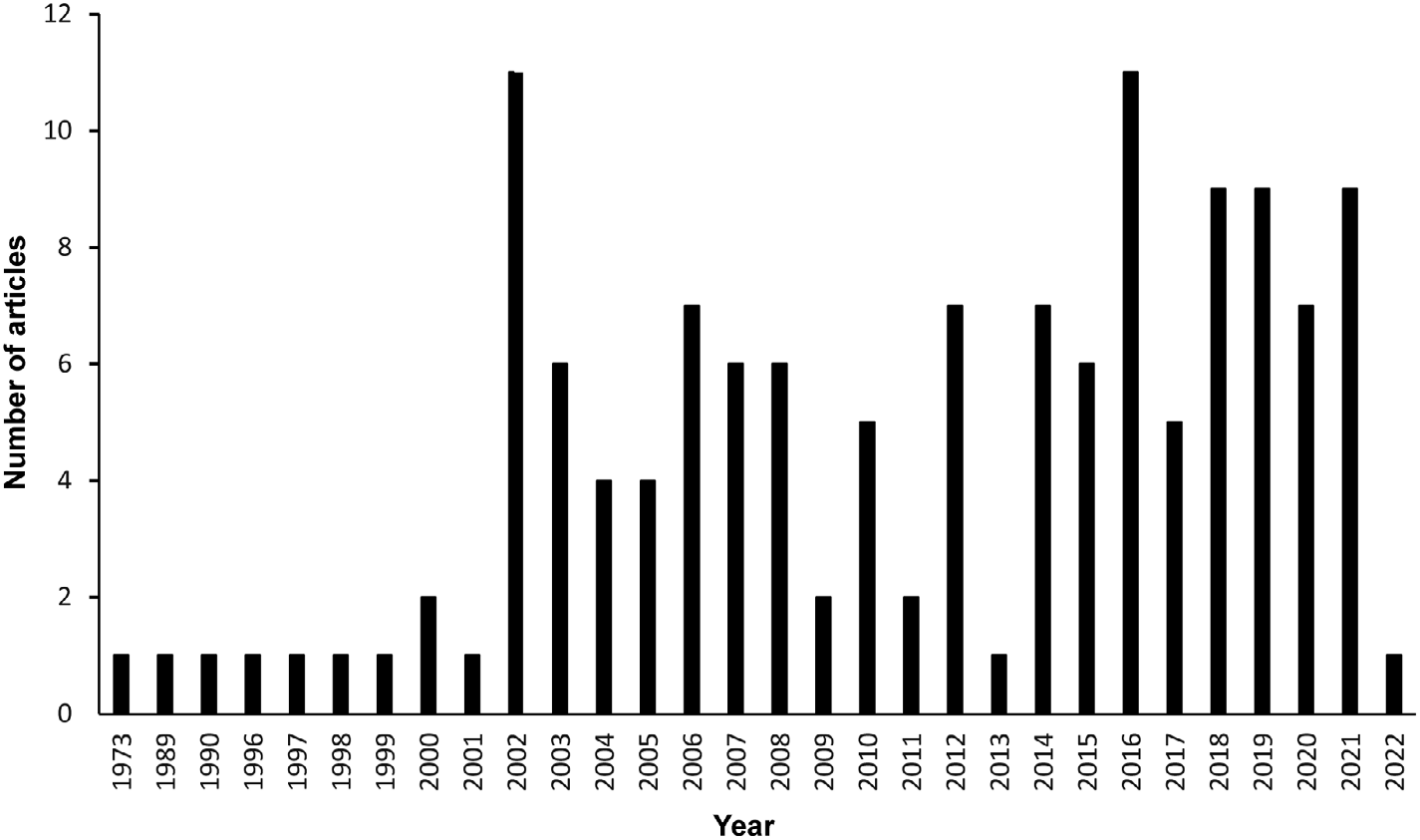
Number of articles published per year regarding humane stunning or stun/killing methods relevant to wild-caught fish between 1973 and 15th February 2022.

Up until the year 2000, a maximum of one primary research study per year was found on humane stunning or stun/killing methods of relevance to wild-caught finfish species. Since 2000, the overall number of articles published has remained below ten per year in all but two years (Figure 2), suggesting that the topic is under-researched. The majority of the research has been published in peer-reviewed journals (n = 125), followed by academic theses (n = 5), conference papers (n = 4) and reports (n = 1).

### Study designs

Study designs were highly heterogenous, sometimes including multiple variables (e.g. pre-slaughter stressors) in addition to the humane stunning or stun/killing methods under investigation (n = 38). Three types of study design were identified: -quasi-experimental, where the study had a control but no randomisation (n = 106); observational studies with no control (n = 95); and randomised controlled trials (n = 22). Methodology was sometimes poorly reported, making interpretation and meta-data extraction of study design challenging.

The majority of studies were conducted under laboratory conditions (n = 151), followed by studies carried out on fish farms or fish slaughter/processing sites (n = 65). Very few studies were conducted onboard fishing vessels (n = 7).

A total of 104 studies reported approval regarding animal welfare for the experimental procedures carried out and/or stated experiments were designed following guidance on the care and use of experimental animals.

### Species investigated

Primary research on humane stunning methods was captured for 33 species of finfish (18 marine species, ten freshwater species and five diadromous species), that are known to be caught in the wild on a commercial scale, and that are intended for consumption by humans and/or animals (Table 2). Around half of the species included in the review are produced in much greater tonnage on a global scale in aquaculture than are caught in the wild (Table 2), and 95% of the studies were carried out on farmed fish, not fish caught directly from the wild (Table 2). The most researched species were rainbow trout and Atlantic salmon, followed by Nile tilapia (*Oreochromis niloticus*); all primarily farmed fish.

**Table 2.** Number of studies included in the review for each species of finfish (farmed or wild-caught) for which humane stunning or stun/killing methods have been tested, presented alongside global wild-capture and aquaculture production statistics (in tonnes), and estimated (upper and lower millions) numbers of wild-caught individuals for the year 2019

English common name^1^	Scientific name^1^	Studies conducted on farmed or wild-caught fish	Aquaculture production 2019 (t)^2^	Global wild-capture production 2019 (t)^2^	Estimated numbers of wild-caught individuals for 2019 in lower-upper millions^3^	Number of studies included in the review
Arctic char	*Salvelinus alpinus*	Farmed	8,479	305	0.07–0.67	5
Atlantic bluefin tuna	*Thunnus thynnus*	Farmed	12,130	30,707	0.02–0.12	1
Atlantic cod	*Gadus morhua*	Farmed & wild	900	1,130,626	282.66 –1413.28	10
Atlantic halibut	*Hippoglossus hippoglossus*	Farmed	1,609	10,237	0.49*	1
Atlantic herring	*Clupea harengus*	Wild	0	1,559,021	2598.37–15590.21	1
Atlantic mackerel	*Scomber scombrus*	Wild	0	86,900	191.41–223.39	2
Atlantic salmon	*Salmo salar*	Farmed	2,615,962	1,807	0.41*	30
Bastard halibut	*Paralichthys olivaceus*	Farmed	45,320	11,007	1.75–1.82	1
Channel catfish	*Icalurus punctatus*	Farmed	453,511	966	0.48*	1
Chilean jack mackerel	*Trachurus murphyi*	Wild	0	657,354	657.35–3286.77	1
Cobia	*Rachycentron canadum*	Farmed	48,163	15,316	0.8–2.41	4
Common carp	*Cyprinus carpio*	Farmed	4,411,900	129,468	8.6–64.73	11
Common sole	*Solea solea*	Farmed	11	28,370	29.22–1216.61	7
European eel	*Anguilla anguilla*	Farmed	5,496	4,140	1.18*	15
European seabass	*Dicentrarchus labrax*	Farmed	263,215	5,206	0.52–17.35	13
Gilt-head bream	*Sparus aurata*	Farmed	258,754	8,258	1.32–13.98	6
Grass carp	*Ctenopharyngodon idellus*	Farmed	5,728,383	24,896	0.68–0.84	3
Haddock	*Melanogrammus aeglefinus*	Wild	0	315,457	175.25–350.50	3
Japanese jack mackerel	*Trachurus japonicus*	Wild	800	188,199	2227.51–12335.99	1
Japanese seabass	*Lateolabrax japonicus*	Farmed	184,020	7,995	No data	1
Japanese yellowtail/amberjack	*Seriola quinqueradiata*	Farmed	135,600	No data but evidence of wild-capture^4^	No data	1
Nile tilapia	*Oreochromis niloticus*	Farmed	4,590,292	281,644	140.82*	16
North African catfish	*Clarias gariepinus*	Farmed	235,580	74,583	9.04–13.78	10
Pacu	*Piaractus mesopotamicus*	Farmed	15,606	33	No data	5
Pike-perch	*Sander lucioperca*	Farmed	3,171	23,145	19.28–158.90	2
Rainbow trout	*Oncorhynchus mykiss*	Farmed	916,540	1,356	0.27–2.71	39
Red seabream/ Red porgy	*Pagrus major*	Farmed	2,933	11,706	17.74*	3
Sea (Brown) trout	*Salmo trutta*	Farmed	3,252	3,696	0.61*	2
Silver carp	*Hypophthalmichthys molitrix*	Farmed	4,827,720	34,198	2.67*	6
South American catfish	*Rhamdia quelen*	Farmed	10	16	No data	6
Tench	*Tinca tinca*	Farmed	792	1,016	0.26*	2
Turbot	*Scophthalmus maximus*	Farmed	77,710	5,972	0.53–2.89	12
Yellowtail amberjack	*Seriola lalandi*	Farmed	407	2,969	0.20–0.67	2

1 ASFIS - Aquatic Sciences and Fisheries Information System common and scientific species name https://www.fao.org/fishery/en/collection/asfis/en.

2 FAO Global Fishery and aquaculture production statistics v. 2021: Global aquaculture production - Quantity (1950-2019) reported in tonnes live weight = 1,000kg; Global capture production (1950-2019) reported in tonnes live weight = 1,000kg. Data rounded to the nearest whole number (FAO 2021).

3 Estimated numbers of individuals in lower and upper millions, calculated using FAO capture tonnage divided by lower and upper ‘estimated mean weights’ for each species, data source Alison Mood Fishcount.org.uk. Data rounded to 2 decimal places. Where estimated mean weights or FAO capture statistics are not available, data is presented as unavailable in the table. For some species there is only one estimated mean weight (i.e. no upper and lower value) as marked by an asterisk (*).

4 Evidence for wild-capture of Japanese amberjack: https://seafood-guide.wwf.org.hk/en/node/995;https://www.trademodo.com/resources/seafood-guide/japanese-amberjack-species-overview/;https://www.fao.org/fishery/en/culturedspecies/seriola_quinqueradiata (FAO state that wild-caught amberjack often command a higher price than farmed, although farmed fish are more commonly sold than wild-caught fish).

Table 2 shows that some typically wild-caught species such as, Atlantic herring (*Clupea harengus*), and Japanese jack mackerel (*Trachurus japonicus*), are caught in large tonnage on a global scale and also comprise of a large number of individuals captured per year. Research for humane stunning or stun/killing of these species is limited especially when considering the magnitude of an animal welfare problem in terms of numbers of individual fish affected (Table 2)

Only 12 studies (reported in six articles), tested humane stunning or stun/killing methods on fish caught directly from the wild (Table 2). The species investigated were: Atlantic cod (*Gadus morhua*) (Kristoffersen *et al.* 2006; Lambooij *et al.* 2012), Atlantic herring (Nordgreen *et al.* 2008), Atlantic mackerel (*Scomber scombrus*) (Anders *et al.* 2019), Chilean jack mackerel (*Trachurus murphyi*) (Lyu *et al*. 2015), haddock (*Melanogrammus aeglefinus*) (Lambooij *et al*. 2012) and Japanese jack mackerel (Mishima *et al.* 2005). Out of these 12 studies, only seven (reported in two articles) were conducted onboard fishing vessels, for Atlantic cod and haddock (Lambooij *et al.* 2012) and Chilean jack mackerel (Lyu *et al.* 2015).

### Geographical distribution of research

**Figure 3.**
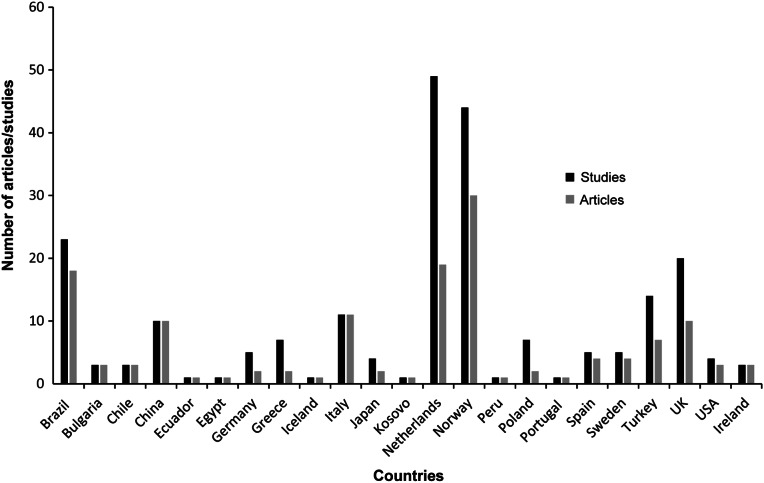
Geographical distribution of origin of research regarding humane stunning or stun/killing methods of relevance to wild-caught fish. Articles may contain one or more study.

Primary research studies originated from 23 countries and the vast majority were conducted in geographical Europe, most notably in The Netherlands and Norway. Whilst the number of studies from some countries was high, this often corresponded to a smaller number of articles published (Figure 3).

**Figure 4.**
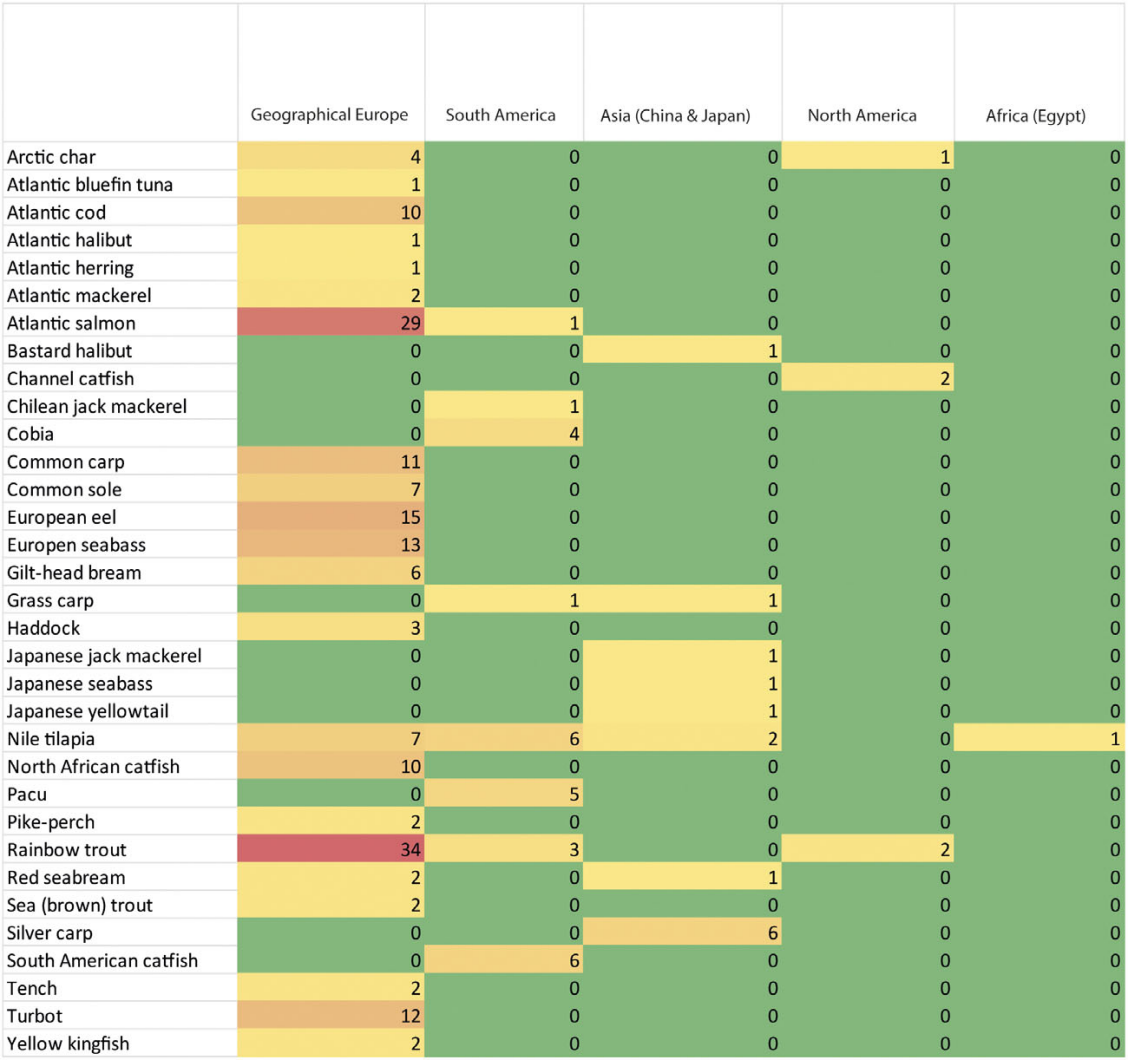
Number of humane stunning or stun/killing research studies conducted in geographical Europe, South America, Asia, North America and Africa for each fish species included in the review.

In geographical Europe, humane stunning or stun/killing methods have been researched for a wide range of species, both native and non-native to European waters, whereas research has been conducted on fewer species in South America (Brazil, Chile, Ecuador and Peru), Asia (China and Japan), North America (USA), and Africa (Egypt) (Figure 4).

### Outcomes measured

The majority of studies investigated the effect of humane stunning or stun/killing methods solely on fish welfare (n = 89), followed by studies investigating the impact of stunning method on flesh quality (n = 75), and on both fish welfare and flesh quality (n = 59). No evidence was captured for other outcomes considered for inclusion in this review including studies investigating: feasibility or economic viability of the use of stunning for wild-caught fish; likelihood of uptake or cost implications of the use of stunning on product price; and refining the process between capture and application of stunning method to minimise suffering prior to stunning.

### Humane stunning or stun/killing methods researched

The most researched humane stunning method was electrical (n = 168), followed by percussive (n = 72), spiking (n = 19) and captive needle to inject compressed air into the brain (n = 7).

Of the 168 studies investigating electrical stunning, 88 investigated in-water electrical stunning, and 50 dry electrical stunning. One study was described as semi-dry electrical stunning, 14 did not make it clear whether the electrical stunning was dry or in-water, and 19 used alternative electrical methods to stun fish. (Note the total number of studies is greater than 168 because some studies investigated more than one method). For electrical stunning a mixture of experimental stunners (~57% of studies) and commercially available stunners (~34%), (often modified for research purposes) were used. In some cases, limited or no information was reported about the type of stunner used.

Seventy-two studies investigated percussive stunning. Of these, 65 were about manual percussive stunning. Instruments used included: wooden clubs, a hand-held, non-penetrative captive-bolt gun, back of a knife, hammer, and non-specified priests. In 30 studies, the type of instrument to perform the manual percussive stun was unreported. Five studies investigated automated machine percussive stunning, all of which tested or modified commercially available machines. The type of percussive stun was unclear in two studies.

Table 3 summarises the humane stunning or stun/killing method and outcome investigated (welfare or flesh quality) for finfish species. In-depth detail of the stunning methods researched, including parameters tested, can be found in the accompanying database (Appendix 2, worksheet 2). These studies illustrate that species-specific humane stunning parameters/protocols are required for each stunning method to ensure welfare and product quality standards. Furthermore, for in-water electrical stunning of diadromous species that inhabit different water environments, more than one protocol may be required. This is because differences in water conductivity lead to differences in the voltage and current which need to be applied to the water in order to stun the fish (Lines & Kestin 2004). This should be considered in the future development of protocols for these species. When considering the number of finfish species that are wild-caught on a global commercially scale, this review clearly shows that more research is needed to determine humane stunning parameters for a wider range of wild-caught species, their environments and stunning methods.

**Table 3.** Summary of humane stunning or stun/killing methods researched and outcomes measured for each finfish species, where ● denotes studies investigating the effect of humane stunning or stun/killing method on fish welfare, and ○ denotes studies investigating the effect of humane stunning or stun/killing method on fish quality

English common name^1^	Species name^1^	Percussive unclear	Manual percussive	Automated percussive	Dry electrical	Semi-dry electrical	In-water electrical	Electrical unclear	Other electrical	Spiking	Captive needle
Arctic char	*Salvelinus alpinus*		○ ●		●		● ○				
Atlantic bluefin tuna	*Thunnus thynnus*								○ harpoon		
Atlantic cod	*Gadus morhua*		○		● ○		● ○				
Atlantic halibut	*Hippoglossus hippoglossus*	○									
Atlantic herring	*Clupea harengus*						● ○				
Atlantic mackerel	*Scomber scombrus*				● ○						
Atlantic salmon	*Salmo salar*		● ○	● ○	● ○		●	●		●○	
Bastard halibut	*Paralichthys olivaceus*									○	
Channel catfish	*Icalurus punctatus*								○ unclear		
Chilean jack mackerel	*Trachurus murphyi*		○								
Cobia	*Rachycentron canadum*						● ○	● ○			
Common carp	*Cyprinus carpio*		○ ●		●		● ○				●
Common sole	*Solea solea*				●						
European eel	*Anguilla anguilla*						● ○	●	● tongs		●
European seabass	*Dicentrarchus labrax*		○			● ○	○ ●	○		● ○	
Gilt-head bream	*Sparus aurata*		○				○ ●	●		●○	
Grass carp	*Ctenopharyngodon idellus*		○				○				
Haddock	*Melanogrammus aeglefinus*				●						
Japanese jack mackerel	*Trachurus japonicus*									○	
Japanese seabass	*Lateolabrax japonicus*		○								
Japanese yellowtail	*Seriola quinqueradiata*									○	
Nile tilapia	*Oreochromis niloticus*		● ○				●	● ○	● ○ net	● ○	
North African catfish	*Clarias gariepinus*		●		●		● ○	●	● tongs		●
Pacu	*Piaractus mesopotamicus*						● ○				
Pike-perch	*Sander lucioperca*				●						
Rainbow trout	*Oncorhynchus mykiss*	○	● ○		○ ●		● ○	● ○	● ○ tongs	○ ●	
Red seabream/ Red porgy	*Pagrus major*						○ ●			○ ●	
Sea (Brown) trout	*Salmo trutta*		○				○ ●			●	
Silver carp	*Hypophthalmichthys molitrix*		○ ●								
South American catfish	*Rhamdia quelen*						○●				
Tench	*Tinca tinca*		○ ●		○ ●						
Turbot	*Scophthalmus maximus*		● ○		●		● ○	○	○ ● tongs		
Yellowtail amberjack	*Seriola lalandi*				●						

1 ASFIS - Aquatic Sciences and Fisheries Information System common and scientific species name https://www.fao.org/fishery/en/collection/asfis/en.

Thirty-eight articles investigated potentially humane stunning methods (Appendix 2, worksheet 4) but the feasibility of using these techniques in a wild-capture setting was not tested. Evidence for potentially humane methods was not searched for specifically and therefore this is unlikely to be a comprehensive list of research carried out on the subject. Potentially humane methods reported included: carbon monoxide, nitric oxide and nitrogen-saturated water, and the use of sedatives including AQUI-S, clove oil, *Lippia alba*, 2-phenoxyethanol. Whilst authors suggest that some of these methods potentially offer benefits for some species in terms of fish welfare and/or flesh quality, they also highlighted limitations, including workplace dangers such as for carbon monoxide and nitric oxide, which are toxic to humans, and impacts on taste and smell of the product from chemical sedatives. One study investigated supercooled brine to stun wild fish. However, the authors reported that it was not possible to confirm the extent to which the fish were stunned by the treatment and that stunning fish in under half a second would not be achievable using ultra-low temperatures (Stormo *et al.* 2022). This study was published and identified after the main searches for literature had been conducted.

### Methods to assess fish welfare

Table 4 summarises the methods used to assess the welfare of different fish species when applying different humane stunning methods. Four main welfare assessment methods were described in the collated research: behavioural/visual indicators, EEG, electrocardiogram (ECG) and blood and/or blood plasma stress markers (e.g. plasma levels of cortisol). These methods were used alone or in combination.

**Table 4.** Summary of primary research methods used to assess the welfare of fish species when applying different humane stunning or stun/killing methods, where ● denotes behavioural indices; ■denotes electroencephalogram (EEG); ▲ denotes electrocardiogram (ECG); ◊ denotes blood or blood plasma indicators of stress

English common name^1^	Species name^1^	Number of studies	Manual Percussive	Automated Percussive	Dry Electrical	Semi-dry Electrical	In-water Electrical	Electrical Unclear	Other Electrical	Spiking	Captive Needle
Arctic char	*Salvelinus alpinus*	4	●◊		● ◊		● ◊				
Atlantic cod	*Gadus morhua*	7			● ■ ▲ ◊		● ■ ▲				
Atlantic herring	*Clupea harengus*	1					●				
Atlantic mackerel	*Scomber scombrus*	2			●						
Atlantic salmon	*Salmo salar*	13	● ■◊	● ■▲	● ■▲		■▲	●■		●■	
Cobia	*Rachycentron canadum*	3					●	●			
Common carp	*Cyprinus carpio*	8	● ■ ▲ ◊		●◊		● ■ ▲ ◊				● ■ ▲
Common sole	*Solea solea*	7			● ■▲						
European eel	*Anguilla anguilla*	14					● ■ ▲	●■	● ■ ▲		● ■ ▲
European seabass	*Dicentrarchus labrax*	10				●	● ■ ▲ ◊			◊	
Gilt-head bream	*Sparus aurata*	2					◊	●		◊	
Haddock	*Melanogrammus aeglefinus*	3			● ■ ▲						
Nile tilapia	*Oreochromis niloticus*	13	● ▲◊				● ■ ▲ ◊	◊	●	● ■ ▲	
North African catfish	*Clarias gariepinus*	10	● ■		■ ▲		● ■ ▲	● ■	● ■ ▲		● ■ ▲
Pacu	*Piaractus mesopotamicus*	4					● ◊				
Pike-perch	*Sander lucioperca*	2			● ■ ▲						
Rainbow trout	*Oncorhynchus mykiss*	26	● ■ ▲ ◊		● ◊		● ■◊	● ■◊	●	●	
Red seabream/ Red porgy	*Pagrus major*	2					◊			◊	
Sea (Brown) trout	*Salmo trutta*	1					◊			◊	
Silver carp	*Hypophthalmichthys molitrix*	1	●								
South American catfish	*Rhamdia quelen*	4					●				
Tench	*Tinca tinca*	1	●◊		●◊						
Turbot	*Scophthalmus maximus*	7	●		● ■ ▲		● ■ ▲		●		
Yellowtail amberjack	*Seriola lalandi*	2			■ ▲						

1 ASFIS - Aquatic Sciences and Fisheries Information System common and scientific species name https://www.fao.org/fishery/en/collection/asfis/en.

For a stunning method to be considered acceptable, loss of consciousness must occur immediately, and last until death occurs (EFSA 2004). One hundred and eighteen studies measured onset and duration of unconsciousness: 64 studies used EEG to assess the state of fish consciousness and 53 studies only used behavioural/visual indicators. Fifty-two out of the 64 studies that used EEG also monitored the electrical activity of the heart using ECG. EFSA recommends unconsciousness is established post-stunning using neurological measures of brain electrical activity (EFSA 2018). The use of EEG is technically challenging and behavioural/visual indicators of consciousness are often seen as a more readily obtainable alternative, especially in studies outside of a laboratory setting (Anders *et al.* 2019; Hjelmstedt *et al.* 2022). It is not surprising, therefore, that the majority of studies using EEG collated in this review, were conducted in a laboratory (n = 56). Only one article, reporting four studies, used EEG onboard a research fishing vessel: Lambooij *et al.* (2012), investigated dry electrical stunning of Atlantic cod and haddock, using a modified commercial stunner. The authors recommended a stun of 52 Vrms (coupled AC/DC current) for more than 3 s, followed by a throat cut to kill the fish whilst still unconscious, as quickly as possible after fish are brought onboard.

Examples of studies where only behavioural/visual indicators have been used to assess state of consciousness, include: Nordgreen *et al.* (2008) who investigated electric field strengths and current durations on the welfare of Atlantic herring. Herring captured using purse seine were transferred to sea cages in Norway for three months and fed a commercial halibut feed to mitigate the potential effects of maturity and capture fatigue. Fish were stunned with an experimental stunner using 50-Hz AC in seawater and exposed to electric field strengths ranging from 16 to 142 V m^–1^ and current durations from 1 to 12 s. Duration of unconsciousness was defined by absence of opercular ventilation until opercular ventilation is regained. The threshold electric field strength required to stun all of the fish to unconsciousness was 33 V m^–1^ for 1 s. Anders *et al.* (2019) assessed the welfare of wild-caught Atlantic mackerel that were dry electrically stunned using a commercial dry electrical stunner (STANSAS, Seaside A/S, Stranda, Norway). Fish were passively attracted to aquaculture cages in Norway and caught using barbless handlines before being transported to the stunner. Fish were placed by hand, head-first, into the stunner (AC/DC supply ≈ 110 Vrms for 5 s), then transferred to ice slurry. Consciousness index scores were based on behavioural indices, and assumed unconsciousness was maintained throughout the post-stunning chilling treatment in all fish. All mackerel were assumed to have died while still unconscious due to their oxyphilic nature and high heat loss of these relatively small fish. The authors suggested electrical stunning followed by immersion in ice slurry to be suitable for this species but recommended more research to confirm by EEG that the behavioural/reflex indicators used are an accurate method of determining mackerel consciousness/death. These authors also suggested that the effectiveness of the protocol may be dependent upon the fat content of the fish, which is seasonal.

Studies included in the review highlighted the requirement for species-specific protocols to assess state of consciousness when evaluating stunning methods. Visual/behavioural indicators alone cannot always be relied upon as an accurate measure of consciousness (e.g. Gräns *et al.* 2016; Rucinque *et al.* 2021). A more reliable way of assessing consciousness may be to correlate indicators with brain activity, but is perhaps not possible for all indicators and species. For example, Hjelmstedt *et al.* (2022), determined no clear relationship between presence and absence of ventilation and visual evoked responses (VERs) following electrical stunning of rainbow trout. These authors also found that the presence of an epileptic-like seizure following electrical stun does not guarantee a prolonged absence of VERs, and that VERs can return before the end of the seizure. As both presence of a seizure and absence of VERs have been used independently as indicators of unconsciousness, the authors recommended further evaluation of the reliability of neurophysiological indicators of unconsciousness when validating methods to stun fish. Hjelmstedt *et al.* (2022) also recommended research to better understand how sensations of fear, pain, distress, and anxiety relate to the presence and absence of different indicators of consciousness, including VERs and ventilation.

Thirty-six studies measured blood and/or blood plasma stress markers, to provide an indication of fish stress at the time of stunning or stun/killing, and the implications for welfare and or flesh quality. Seventeen out of the 36 studies only assessed the impact of stunning method on fish welfare using these markers.

In-depth detail of all the studies that assessed fish welfare can be found in the accompanying database (Appendix 2, worksheet 2).

### Application of humane stunning or stun/killing methods to wild-caught fish

The development and implementation of humane stunning technologies originated in the aquaculture sector, so it is unsurprising that most of the research into humane stunning of relevance to wild-caught fish, has been carried out on farmed fish (211 out of 223 studies), mainly under controlled laboratory conditions (n = 149) but sometimes in a farm setting (n = 62). It is likely that this research will help to inform humane stunning of the same fish species captured in a wild setting. Conditions and challenges in wild-capture fisheries are, however, very different to those on a farm or in a laboratory, which may have implications for fish welfare, product quality and practical implementation. For example: wild fish are subject to different pre-slaughter stressors compared to farmed fish, especially during capture (e.g. Waley *et al.* 2021); parameters for effective stunning (automated percussive and electrical) rely on fish being of uniform size (e.g. HSA 2018), which is often not the case when harvesting wild fish; there are practical issues associated with stunning large volumes of fish in dynamic conditions and a short space of time (e.g. Lyu *et al.* 2015; Anders *et al.* 2019), including removing by-catch (van de Vis 2020) and correctly orientating the target species into the automated stunner (e.g. Anders *et al.* 2019).

In this review, the only studies conducted onboard boats were those by Lambooij *et al.* (2012) and Lyu *et al.* (2015), the former on a research vessel and the latter on a commercial vessel. Parameters determined by Lambooij *et al.* (2012) for electrical stunning of wild Atlantic cod were lower than those required for farmed cod (107 Vrms for 15 s), determined by Erikson *et al.* (2012), using the same modified commercial stunner. Lambooij *et al.* (2012) hypothesised that impedance of the skin is lower for cod caught at sea, compared to farmed cod possibly due to skin damage and/or to stress caused by trawling. Lyu *et al.* (2015), reported that manual percussive stunning of Chilean jack mackerel was difficult for fishermen to manipulate, and it was easy to damage fish appearance and make fish bleed. This review highlights the requirement for more scientific testing of stunning methods in wild-capture commercial settings, to help ensure: practicality; that welfare standards are met; and quality of product is not affected.

The authors of this review are aware that: (1) humane stunning technology (percussive and dry or semi-dry electrical) developed in the aquaculture sector, is being used on a small scale, for a limited number of species, in commercial wild-capture fisheries. To their knowledge, however, no scientific validation of these methods in a commercial wild-capture setting has been conducted, particularly regards fish welfare. Moreover, it has been reported in the scientific literature that, in some cases, electrical stunners in wild-capture fisheries are being used to electro-immobilise fish rather than render fish unconscious (Erikson *et al.* 2021). This is done to make rapid bleeding of live fish more feasible and improve the health and safety of fishermen, since the fish are easier and safer to handle during the bleeding operation (Erikson *et al*. 2016, 2021). However, fish that are electro-immobilised but still conscious remain sensible to fear, distress and suffering at the of killing; (2) commercial (e.g. Pyne-Carter 2021) and academic research (H van de Vis, personal communication 2022) is being conducted to develop in-water, continuous-flow electric stunners for use in wild-capture fisheries. These types of stunner reduce fish handling and exposure to air which cause stress in fish (van de Vis *et al.* 2017), and in some cases overcome practical issues associated with stunning fish of different sizes and in large volumes (Pyne-Carter 2021). The academic research plans to: test the stunning method for its effectiveness and suitability of the vessel’s fish-handling procedures and well-being of the crew, and how best to communicate improved fish welfare to consumers (H van de Vis, personal communication 2022). However, at the time of writing, these technologies have not been implemented or scientifically tested onboard wild-capture fishing vessels.

### Flesh quality

Whilst a particular stunning method may improve the welfare of a certain species, in order for the method to be implemented in commercial practice, it must not compromise flesh quality. Many of the studies included in this review considered only fish welfare or flesh quality not both.

A wide range of flesh quality outcomes were investigated in the studies included in this review, from: simple examination of fillets for haematomas or spinal column fractures, to more in-depth experiments to examine the impact on quality parameters over time and under different storage conditions. It is widely recognised in certain sectors of the aquaculture industry that humane stunning can improve flesh quality (HSA 2018). This is evidenced by the studies in this review conducted on species that are more often farmed than wild-caught. When considering studies of species that are only wild-caught, the impact of stunning method on flesh quality had mixed results: Anders *et al.* (2019) found no evidence of spinal damage or blood haematomas post-filleting, in Atlantic mackerel that were dry electrically stunned. The authors suggested, however, that more research is required to investigate flesh quality implications associated with the depletion of energy reserves following electrical stunning. Nordgreen *et al.* (2008), on the other hand, concluded that although in-water electrical stunning would improve the welfare of Atlantic herring landed alive, and that the method could be adapted to fishing vessels, fillet quality was so negatively affected that it would not be deemed acceptable to the herring fillet industry. Lyu *et al.* (2015) compared the impact of manual percussive stunning to asphyxiation in ice water or air on the quality of Chilean jack mackerel during refrigerated onboard storage. The increase in total volatile basic nitrogen (TVB-N), trimethylamine (TMA) and 2-thiobarbituric acid reactive substances (TBARS) values with storage time, was lower in mackerel asphyxiated in ice water and these fish also had a better sensory score. The authors concluded that killing method of asphyxia in ice-water, regarded as inhumane by the OIE (2019), could maintain better quality of fish during refrigerated storage.

In-depth detail of all the studies that assessed flesh quality can be found in the accompanying database (Appendix 2, worksheet 2).

## Recommendations for future research

This review has identified multiple knowledge gaps in the scientific evidence base. Recommendations for future research include:

- Determining humane stunning parameters for a wider range of species, their environments and stunning methods. A large number of finfish species are commercially wild-caught on a global scale and so there is a need to prioritise species for future research.
- Developing species-specific protocols for a wider range of wild-caught fish that can be used to accurately assess state of consciousness when evaluating stunning methods.
- More use of brain activity measurements to accurately verify loss of consciousness on application of the intended stunning method.
- Correlation of species-specific behavioural/visual indicators to brain activity that can be used to evaluate humane stunning in commercial practice where it is often difficult to obtain EEG.
- Investigating whether loss of consciousness resulting from humane stunning in controlled studies can be achieved in a commercial context (i.e. onboard fishing vessels) using species-specific behavioural indicators and/or measures of brain activity.
- Scientific verification of humane stunning methods/technologies (e.g. commercial dry stunners) that are currently in use onboard commercial fishing vessels to ensure welfare standards are being met.
- Studies that consider the effect of stunning method on both fish welfare and product quality.
- Further development of humane stunning technology for use onboard boats which minimises handling and exposure of fish to air prior to stunning and that is capable of stunning large volumes of fish of non-uniform maturity/size and catches that may contain mixed species.
- Consideration of factors in wild-capture fisheries that may affect efficacy of stunning method. For example, impact of seasonal body fat content on efficacy of electrical stunning.
- Research to refine the process between capture and application of stunning method to minimise suffering prior to stunning.
- Feasibility studies to investigate the practical challenges associated with humane stunning methods/technology onboard fishing vessels.
- Cost/benefit analysis of humane stunning in wild-capture fisheries, including cost implications on product price.
- Studies to better understand how human behaviour in the fish supply chain (e.g. fishermen, processors, retailers and consumers) influences uptake of stunning and demand for humanely stunned fish.

## Limitations of the review and evidence base

Searches for literature were only carried out in English and Spanish and therefore some relevant articles may have been missed. Non-English and Spanish language texts (n = < 40) were not translated and translation may have extended the evidence base. Unpublished research may be under-represented in this review, particularly where it is not available online or is commercially sensitive. No formal critical appraisal of studies or quantitative analyses were carried out in this review and any conclusions drawn by study authors come with the caveat that risk of bias has not been assessed.

Reporting of methodology and results was sometimes unclear. We recommend that authors of future studies report methodology and results more clearly to enable readers to judge the validity of the study results and other scientists to repeat the study during efforts to validate the findings. Furthermore, use of terminology by study authors was inconsistent. Multiple terms were used to describe humane stunning methods and insensibility post-stunning. For example, electrical stunning was described as, electronarcosis, electro-sedation, electro-stunning. Fish were described as anaesthetised (where anaesthetics were not used), immobilised or desensitised by the stunning method and it was unclear what the definition of these terms were. We therefore also recommend that scientists and industry around the world use standard terminology.

## Animal welfare implications and conclusion

The slaughter of wild-caught fish for food and feed, using methods that result in poor fish welfare, is estimated to impact over a trillion individuals worldwide each year. Despite the enormity of this animal welfare issue, there is a paucity of scientific research underpinning humane stunning or stun/killing, as a means of improving wild-fish welfare at slaughter. Multiple knowledge gaps exist, where future research needs to be prioritised.

From an animal welfare perspective, it is critical to ensure that there is loss of consciousness on application of the stunning method, and that the fish remain unconscious long enough to avoid recovery before subsequent death. This review calls attention to the need for research to develop species-specific protocols, to accurately assess states of consciousness on application of the stunning method, and how this can be evaluated in commercial settings.

Applied research is also needed to assess the practical feasibility of implementing humane stunning or stun/killing methods in a commercial context, for different fish species and types/sizes of fishery. Moreover, future research should investigate drivers to uptake of humane stunning or stun/killing, in terms of the wider benefits to fishermen.

In conclusion, this review supports the need for funding to carry out further research including commercial feasibility trials, to enable uptake of humane stunning or stun/killing in commercial wild-capture fisheries to progress.

## Supporting information

James et al. supplementary material 1 James et al. supplementary material

James et al. supplementary material 2 James et al. supplementary material

## Supplementary material

The supplementary material for this article can be found at http://doi.org/10.1017/awf.2023.30.

## References

[r1] Anders N, Roth B, Grimsbø E and Breen M 2019 Assessing the effectiveness of an electrical stunning and chilling protocol for the slaughter of Atlantic mackerel (*Scomber scombrus*). PLoS ONE 14(9): e0222122. 10.1371/journal.pone.0222122 31483840 PMC6726217

[r2] Animal Protection Ordinance (AniPO) 455.1 2008 *Animal Protection Ordinance (AniPO) 455.1 of 23 April 2008 (status as at 1 March 2018).* Switzerland.

[r3] Braithwaite V 2010 Do Fish Feel Pain? Oxford University Press: Oxford, UK.

[r4] Broom DM 2016 Fish brains and behaviour indicate capacity for feeling pain. Animal Sentience 1(3): 4. 10.51291/2377-7478.1031

[r5] Collaboration for Environmental Evidence 2018 *Guidelines and Standards for Evidence synthesis in Environmental Management. Version 5.0.* http://www.environmentalevidence.org/information-for-authors

[r6] Erikson U, Lambooij B, Digre H, Reimert HGM, Bondø M and van der Vis H 2012 Conditions for instant electrical stunning of farmed Atlantic cod after de-watering, maintenance of unconsciousness, effects of stress, and fillet quality - a comparison with AQUI-STM. *Aquaculture* 324/325: 135–144. 10.1016/j.aquaculture.2011.10.011

[r7] Erikson U, Digre H and Grimsmo L 2016 Electrical immobilisation of saithe (*Pollachius virens*): Effects of pre-stunning stress, applied voltage, and stunner configuration. Fisheries Research 179: 148–155. 10.1016/j.fishres.2016.02.017

[r8] Erikson U, Grimsmo L and Digre H 2021 Establishing a method for electrical immobilization of whitefish on board fishing vessels. Journal of Aquatic Food Product Technology 30(6): 694–705. 10.1080/10498850.2021.1931606

[r9] European Food Safety Authority (EFSA) 2004 Opinion of the Scientific Panel on Animal Health and Welfare on a request from the Commission related to welfare aspects of the main system of stunning and killing the main commercial species of animals. EFSA Journal 45: 1–29. 10.2903/j.efsa.2004.45

[r10] European Food Safety Authority (EFSA) 2009a General approach to fish welfare and to the concept of sentience in fish Scientific Opinion of the Panel on Animal Health and Welfare. EFSA Journal 954: 1–27. 10.2903/j.efsa.2009.954

[r11] European Food Safety Authority (EFSA) 2009b Species-specific welfare aspects of the main systems of stunning and killing of farmed sea bass and sea bream. Scientific Opinion of the Panel on Animal Health and Welfare. EFSA Journal 1010: 1–52. 10.2903/j.efsa.2009.1010

[r12] European Food Safety Authority (EFSA) 2009c Species-specific welfare aspects of the main systems of stunning and killing of farmed fish: rainbow trout. Scientific Opinion of the Panel on Animal Health and Welfare. EFSA Journal 1013: 1–55. 10.2903/j.efsa.2009.1012

[r13] European Food Safety Authority (EFSA) 2009d Species-specific welfare aspects of the main systems of stunning and killing of farmed carp. Scientific Opinion of the Panel on Animal Health and Welfare. EFSA Journal 1013: 1–37. 10.2903/j.efsa.2009.1013

[r14] European Food Safety Authority (EFSA) 2009e Species-specific welfare aspects of the main systems of stunning and killing of farmed turbot. Scientific Opinion of the Panel on Animal Health and Welfare. EFSA Journal 1073: 1–34. 10.2903/j.efsa.2009.1073

[r15] European Food Safety Authority (EFSA) 2009f Species-specific welfare aspects of the main systems of stunning and killing of farmed Atlantic salmon. Scientific Opinion of the Panel on Animal Health and Welfare. *EFSA Journal 2012*: 1–77. 10.2903/j.efsa.2009.1011 PMC10193664 37213864

[r16] European Food Safety Authority (EFSA) 2009g Species-specific welfare aspects of the main systems of stunning and killing of farmed eels (*Anguilla anguilla*). Scientific Opinion of the Panel on Animal Health and Welfare. EFSA Journal 1014: 1–42. 10.2903/j.efsa.2009.1014

[r17] European Food Safety Authority (EFSA) 2009h Species-specific welfare aspects of the main systems of stunning and killing of farmed tuna. Scientific Opinion of the Panel on Animal Health and Welfare. *EFSA Journal* 1072: 1–53. 10.2903/j.efsa.2009.1072

[r18] European Food Safety Authority (EFSA) 2018 Guidance on the assessment criteria for applications for new or modified stunning methods regarding animal protection at the time of killing. EFSA Journal 16: 1–35. 10.2903/j.efsa.2018.5343 PMC7009557 32625979

[r19] FAO 2021 Fishery and Aquaculture Statistics. Global capture production 1950-2019 (FishstatJ). *FAO Fisheries and Aquaculture Department* [online]. Rome. Updated 2021. www.fao.org/fishery/statistics/software/fishstatj/en

[r20] Gräns A, Niklasson L, Sandblom E, Sundell K, Algers B, Berg C, Lundh T, Axelsson M, Sundh H and Kiessling A 2016 Stunning fish with CO_2_ or electricity: contradictory results on behavioural and physiological stress responses. Animal 10(2): 294–301. 10.1017/S1751731115000750 25959256 PMC4762244

[r21] Grimsbø E 2016 Measuring methods for fish welfare during slaughter based on electrical impedance, EEG, ECG and blood. PhD Thesis, University of Bergen, Norway. https://hdl.handle.net/1956/12226

[r22] Haddaway NR, Macura B, Whaley P and Pullin AS 2017 *ROSES for Systematic Map Protocols Version 1.0.* https://www.roses-reporting.com/systematic-map-protocols

[r23] Hjelmstedt P, Sundell E, Brijs J, Berg C, Sandblom E, Lines J, Axelsson M and Gräns A 2022 Assessing the effectiveness of percussive and electrical stunning in rainbow trout: Does an epileptic-like seizure imply brain failure? Aquaculture 552: 738012. 10.1016/j.aquaculture.2022.738012

[r24] Humane Slaughter Association 2018 *Humane slaughter of finfish farmed around the world.* https://www.hsa.org.uk/downloads/hsafishslaughterreportfeb2018.pdf

[r25] James K, Randall NP and Haddaway NR 2016 A methodology for systematic mapping in environmental sciences. Environmental Evidence 5(1): 1–13.

[r26] James KL, Jayasuriya NS, Herath TK, Lines J, Sneddon LU, Amarasinghe US, Prats Aparicio S and Randall NP 2020 *Evidence for humane stunning in the slaughter of wild-caught fish for food: A Systematic Map Protocol.* Published online on the Systematic Reviews for Animals and Food platform. http://www.syreaf.org/

[r27] Kristoffersen S, Tobiassen T, Steinsund V and Olsen RL 2006 Slaughter stress, post mortem muscle pH and rigor development in farmed Atlantic cod (*Gadus morhua* L). International Journal of Food Science and Technology 41(7): 861–864. 10.1111/j.1365-2621.2005.01149.x

[r28] Lambooij E, Digre H, Reimert HGM, Aursand IG, Grimsmo L and van de Vis JW 2012 Effects of on-board storage and electrical stunning of wild cod (*Gadus morhua*) and haddock (*Melanogrammus aeglefinus*) on brain and heart activity. Fisheries Research 127: 1–8. 10.1016/j.fishres.2012.04.004

[r29] Landis JR and Koch GG 1977 The measurement of observer agreement for categorical data. Biometrics 33(1): 159–174. 10.2307/2529310 843571

[r30] Lines J and Kestin S 2004 Electrical stunning of fish: The relationship between the electric field strength and water conductivity. Aquaculture 241: 219–234. 10.1016/j.aquaculture.2004.07.023

[r31] Lyu F, Huang R, Liu L, Zhou X and Ding Y 2015 Effect of slaughter methods on the quality of Chilean jack mackerel (*Trachurus murphyi*) during refrigerated storage. Journal of Food Science and Technology (Mysore) 52(3): 1742–1747. 10.1007/s13197-013-1114-8 PMC4348266 25745250

[r32] Mishima T, Nonaka T, Okamoto A, Tsuchimoto M, Ishiya T, Tachibana K and Tsuchimoto M 2005 Influence of storage temperatures and killing procedures on post-mortem changes in the muscle of horse mackerel caught near Nagasaki Prefecture, Japan. Fisheries Science 71(1):187–194. 10.1111/j.1444-2906.2005.00947.x

[r33] New Zealand’s Commercial Slaughter Code of Welfare 2018 *New Zealand’s Commercial Slaughter Code of Welfare of 1 October 2018.* https://www.mpi.govt.nz/dmsdocument/46018-Code-of-Welfare-Commercial-slaughter

[r34] Nordgreen AH, Slinde E, Møller D and Roth B 2008 Effect of various electric field strengths and current durations on stunning and spinal injuries of Atlantic herring. Journal of Aquatic Animal Health 20: 110–115. 10.1577/H07-010.1 18783132

[r35] Norway Decree No 1250 2006 *Regulations on slaughterhouses and production facilities for aquaculture animals 2006 Norway. Ministry of Trade and Industry. FOR-2006-10-30-1250.* https://lovdata.no/dokument/SF/forskrift/2006-10-30-1250

[r36] OIE 2019 *Welfare aspects of stunning and killing of Farmed fish for human consumption.* *Chapter 7.3 Aquatic Animal Health Code.* World Organisation for Animal Health (OIE). https://www.woah.org/fileadmin/Home/eng/Health_standards/aahc/current/chapitre_welfare_stunning_killing.pdf

[r37] Papaharisis L, Tsironi T, Dimitroglou A, Taoukis P and Pavlidis M 2019 Stress assessment, quality indicators and shelf life of three aquaculture important marine fish, in relation to harvest practices, water temperature and slaughter method. Aquaculture Research 50(9): 2608–2620. 10.1111/are.14217

[r38] Pyne-Carter N 2021 *Advancements in humane slaughter: Food for thought.* Aquaculture North America virtual webinar, 11th May 2021.

[r39] Robb DHF and Kestin SC 2002a Methods Used to kill fish: Field observations and literature reviewed. Animal Welfare 11: 269–282.

[r40] Rucinque GDSR 2016 Perception on fish sentience, welfare and humane slaughter by Latin American citizens and electrical stunning in pacu and South American catfish. Masters Thesis, Federal University of Paraná, Brazil. https://acervodigital.ufpr.br/handle/1884/43675

[r41] Rucinque DS, Biscaia AGC, Watanabe AL and Molento CFM 2021 Electrical stunning in South American catfish (*Rhamdia quelen*) using direct current waveform: welfare and meat quality. Ciência Rural 51: 8. 10.1590/0103-8478cr20200547

[r42] Sneddon LU and Brown C 2020 Mental capacity of fishes. In Johnson L, Fenton A and Shriver A (Eds.) Neuroethics and Nonhuman Animals. Advances in Neuroethics pp 401–439. Springer: Cham, Switzerland. 10.1007/978-3-030-31011-0_4

[r43] Stormo SK, Hustad A and Tobiassen T 2022 *Ultra-low temperature stunning.* https://nofima.com/results/stunning-fish-with-supercooled-brine-has-potential/

[r44] Van de Vis H 2020 *Wild fish slaughter - capture, loading, storage and slaughter.* Aquatic Animal Welfare Conference 2020 (Virtual), 2–6 November 2020. https://www.youtube.com/watch?v=F5zr_4bfhLI&list=PL9iiB0zl8tofkAJ0ZK2GXg_gQ7Z-8A3ZK&index=21&t=1852s

[r45] Van de Vis H, Bergevoet R, Stokkers R, Schrijver R, Dewar D, van de Braak K and Witkamp S 2017 *Welfare of farmed fish: Common practices during transport and at slaughter.* Final Report for the European Commission SANTE/2016/G2/009 pp 1–186. 10.2875/172078

[r46] Waley D, Harris M, Goulding I, Correira-Mega Pesca Lda M and Carpenter G 2021 *Catching up: Fish welfare in wild capture fisheries.* Eurogroup for Animals report 2021. https://www.eurogroupforanimals.org/files/eurogroupforanimals/2021-01/2021_01_12_eurogroup_for_animals_wild_fisheries_report.pdf

